# Into the abyss: diabetes process of care indicators and outcomes of defaulters from a Canadian tertiary care multidisciplinary diabetes clinic

**DOI:** 10.1186/1472-6963-13-303

**Published:** 2013-08-10

**Authors:** Janine C Malcolm, Julie Maranger, Monica Taljaard, Baiju Shah, Chetna Tailor, Clare Liddy, Erin Keely, Teik Chye Ooi

**Affiliations:** 1 Department of Medicine, University of Ottawa, Ottawa, ON Canada; 2 The Ottawa Hospital Research Institute, Ottawa, ON Canada; 3 Clinical Epidemiology Program, Ottawa Hospital Research institute, Ottawa, ON Canada; 4 Department of Epidemiology and Community Medicine, University of Ottawa, Ottawa, ON Canada; 5 Department of Medicine, University of Toronto, Toronto Canada; 6 Department of Medicine, Sunnybrook Health Sciences Centre, Toronto, ON Canada; 7 Bruyere Research Institute, Ottawa, ON Canada; 8 Department of Family Medicine, University of Ottawa, Ottawa, ON Canada; 9 The Ottawa Hospital, Ottawa, ON Canada; 10 Institute for Clinical Evaluative Sciences, Toronto, ON Canada

## Abstract

**Background:**

Continuity of care is essential for good quality diabetes management. We recently found that 46% of patients defaulted from care (had no contact with the clinic for 18 months after a follow-up appointment was ordered) in a Canadian multidisciplinary tertiary care diabetes clinic. The primary aim was to compare characteristics, diabetes processes of care, and outcomes from referral to within 1 year after leaving clinic or to the end of the follow-up period among those patients who defaulted, were discharged or were retained in the clinic.

**Methods:**

Retrospective cohort study of 193 patients referred to the Foustanellas Endocrine and Diabetes Center (FEDC) for type 2 diabetes from January 1, 2005 to June 30, 2005. The FEDC is the primary academic referral centre for the Ottawa Region and provides multidisciplinary diabetes management. Defaulters (mean age 58.5 ± 12.5 year, 60% M) were compared to patients who were retained in the clinic (mean age 61.4 ± 10.47 years, 49% M) and those who were formally discharged (mean age 61.5 ± 13.2 years, 53.3% M). The chart audit population was then individually linked on an individual patient basis for laboratory testing, physician visits billed through OHIP, hospitalizations and emergency room visits using Ontario health card numbers to health administrative data from the Ministry of Health and Long-Term Care at the Institute for Clinical and Evaluative Sciences (ICES).

**Results:**

Retained and defaulted patients had significantly longer duration of diabetes, more microvascular complications, were more likely to be on insulin and less likely to have a HbA1c < 7.0% than patients discharged from clinic. A significantly lower proportion of patients who defaulted from tertiary care received recommended monitoring for their diabetes (HbA1c measurements, lipid measurements, and periodic eye examinations), despite no difference in median number of visits to a primary care provider (PCP). Emergency room visits were numerically higher in the defaulters group.

**Conclusions:**

Patients defaulting from a tertiary care diabetes hospital do not receive the recommended monitoring for their diabetes management despite attending PCP appointments. Efforts should be made to minimize defaulting in this group of individuals.

## Background

The importance of continuity of care in diabetes management has been well recognized. Continued attendance at multidisciplinary diabetes management programs has been shown to decrease the risk of long-term diabetes complications [1-4], improve the uptake of preventive care, enhance adherence to therapy, increase patient and physician satisfaction [5] and decrease hospitalizations [6] and emergency room visits [7-9]. Despite these benefits, high rates of attrition and loss to follow-up have been reported [10-12]. Defaulting from diabetes clinics may be defined as the failure of a patient to maintain contact with the clinic despite a scheduled follow-up appointment made by the specialist. Defaulting from diabetes clinics is associated with adverse outcomes including the development of significant microvascular disease, worsening of biochemical profile and all-cause mortality [13].

The effect of defaulters on health care utilization is likely significant. Patient care may be negatively impacted by large numbers of patients requiring re-referral for specialized services. Re-referral rates for defaulted patients with diabetes to specialized care have been reported to be as high as 20% [10]. Among elderly patients with diabetes, loss of continuity of care is associated with increased hospitalizations and emergency room visits [6]. To date, studies on patients defaulting from specialty diabetes clinics have focused primarily on clinical outcomes. Understanding the health care utilization of diabetes patients defaulting from specialty care is important in developing strategies to improve continuity of care for patients with type 2 diabetes (DM2).

A local audit of our tertiary care multidisciplinary clinic indicated that 23% of patients referred by primary care providers for diabetes care were discharged back to primary care, while 46% of patients defaulted from clinic [14]. We suspect that the extent of defaulting observed in our centre may be reflective of other tertiary care diabetes clinics.

In this paper, we describe the characteristics, long-term outcomes, and health care utilization of patients who defaulted from clinic and compare them to those who were retained or were discharged back to the care of their primary care provider.

## Methods

The Foustanellas Endocrine and Diabetes Centre (FEDC) in Ottawa, Canada is the academic referral centre for the Ottawa region. It serves a catchment area of over one million people and provides specialized interdisciplinary clinical management, education and self-management support for diabetes. There are approximately 20,000 patient visits per year, of which 20% are new referrals.

A chart audit was conducted at the FEDC by an endocrinology fellow and a diabetes nurse specialist. The first 10 charts were reviewed independently by both auditors and the results compared. A data collection guide was developed based on discrepancies between the two auditors. The remainder of the charts was divided between the two auditors and data were entered electronically into the database using the data collection guide. The auditors met on a regular basis to review items for which interpretation remained unclear after using the data collection guide.

The chart audit population was then individually linked using Ontario health card numbers to health administrative data from the Ministry of Health and Long-Term Care housed at the Institute for Clinical and Evaluative Sciences (ICES). Because of the single-payer universal health care system in Ontario, these data describe all care received by Ontario residents with no missing data or loss to follow-up. The databases used in this study included: the Registered Persons Database, which records health care eligibility and demographic information such as date of death; the discharge abstract database for hospital admission information occurring after the date of final recorded visit with the FEDC during the review period; the National Ambulatory Care Reporting System for emergency department visits occurring after the last recorded visit with the FEDC during the review period; the physician service claims database for family doctor and specialist visits, laboratory tests and renal replacement therapy; and the ICES Physicians Database for location and type of physician practice.

Laboratory investigations done at the Ottawa Hospital were in the OACIS Ottawa Hospital Database. This database was searched by the investigators for all pertinent laboratory results done in the follow-up period for all three groups.

All patients who were first seen by an endocrinologist for management of DM2 between January 1 and June 30, 2005 were included in a retrospective chart review. Of the 923 referred diabetes patients, 226 were excluded due to a clear diagnosis of type 1 diabetes (DM1). A computer-generated, uniform random number was assigned to the remaining 697 patients who were then assessed for eligibility in random sequence until the targeted sample size of 200 was reached. The sample size of 200 was chosen to allow a two-sided 95% Confidence Interval (CI) for a single proportion (e.g., the proportion of patients discharged from the clinic), using the large sample normal approximation, to extend 7.1% from the most conservative proportion of 50%, while allowing for a 5% patient exclusion rate.

Data collected in the FEDC chart review included: demographics, past medical history, medication use, processes of care indicators, outcome of care indicators, number of visits to each diabetes team member (physician, nurse, dietician, social worker), and current status with FEDC (discharged back to PCP, retained, or defaulted). Defaulting was defined as no contact with the clinic for 18 months after a follow-up appointment was ordered by the specialist. Data collection continued until the patient was discharged or had defaulted or until the end of the follow-up period in October 2008, whichever came first.

Individuals were then linked between databases and across time via their Ontario health card number, anonymized using a reproducible encryption algorithm. Processes of care indicators (percent receiving A1c measurements, lipid profiles, serum creatinine, annual eye examination), health care utilization (mean visits to primary care physician) and outcomes (emergency room visits and hospital admissions) were collected.

Demographic information, and first and last visit data information was compared among the three groups. The proportions of patients meeting the 2003 Canadian Diabetes Association (CDA) recommended targets (CDA guidelines) for glycated hemoglobin (A1C ≤ 7%, LDL cholesterol <2.5 mmol/L, and blood pressure ≤ 130/80 were calculated ; the proportions of patients meeting one, two or all three targets were compared among the groups. This study was developed prior to the publication of the 2013 CDA guidelines, at which point some of the targets changed. The 2003 guidelines differ from the 2013 guidelines in a few minor areas. The glycated hemoglobin target remained ≤ 7% for most patients with type 1 and type 2 diabetes; however the 2013 guidelines allow for greater individualization of targets for patients based on their age, duration of diabetes, risk of severe hypoglycaemia, life expectancy, and presence cardiovascular disease. The LDL-cholesterol target has dropped to <2.0 mmol/L based on evidence from a number of large randomized controlled trials published after 2003. The blood pressure targets remained the same. As these changes were minor, the difference in guidelines from 2003 to 2013 does not have a significant effect the interpretation of the results.

This study was approved by the Ottawa Hospital Research Ethics Board.

### Statistical analysis

Using chart review data, the demographic characteristics and first and last visit outcomes of the patient cohort were summarized for defaulters retained and discharged groups. Frequencies and percentages were calculated for categorical variables, means and standard deviations for continuous variables, and medians and interquartile ranges (IQR) for ordinal variables or variables with skewed distributions. Differences in process and clinical outcome measures among the three groups (retained, discharged, and defaulted) were tested for statistical significance using 1-way analysis of variance in the case of continuous variables, chi-squared tests or Fisher’s exact tests for categorical variables, and Kruskal-Wallis tests for ordinal variables or variables with skewed distributions. Because we recognize that multiple testing can lead to spurious statistical significance, we considered all these tests as exploratory. If the overall test among the three groups was significant at α = 0.15, we proceeded to conduct pairwise tests to further delineate differences between the groups. To maintain the familywise error rate of the pairwise comparisons, the Bonferroni-corrected significance level of α = 0.017 was used for these pairwise comparisons. It was expected that some proportion of the items would be incomplete in this chart review. For example, we only recorded retinopathy as present or absent if a test for retinopathy had been indicated in the chart; if no test result were available, this item was recorded as missing. No imputation for missing items was planned; each analysis was based on subjects with non-missing values for the relevant variables.

Using the linked health administrative data, dichotomous process of care, and outcomes of defaulters, the retained and discharged groups were described using point estimates with 95% confidence intervals for proportions using the normal approximation to the binomial distribution, or the exact binomial method in the case of small frequencies.

Outcomes summarized as counts (e.g., number of visits with primary care providers, or number of HbA1C measurements) were described as annual rates, together with 95% Poisson confidence intervals using exact methods or using the normal approximation with continuity correction.

The statistical significance of differences in process of care, and proximal and distal outcomes among the three patient groups were assessed using chi-squared tests for dichotomous variables and one-way analysis of variance for continuous variables.

All analyses were conducted using SAS version 9.1 (Cary, NC).

## Results

Of the original 200 patients, one chart was noted to be entered twice during statistical analysis and was excluded. Of the remaining 199 patients, 2 patients had died and 4 were transferred to another institution. These patients, making up only 3.1% of the total cohort, are not included in further description and analysis. The characteristics of the remaining 193 patients at their first visit to the FEDC are presented in Table 1. Referred patients had a median duration of diabetes of 6 years (IQR 1–12), and lived primarily in an urban setting (90.2%). Macrovascular complications were present in 51 patients (28.3%) and 79 patients (40.9%) had microvascular complications.

**Table 1:** Comparison of first visit characteristics among patients retained, discharged, and defaulted

**First visit characteristics**	**Overall (N = 193)**	**Retained (R) (N = 59)**	**Discharged (D/C) (N = 46)**	**Defaulted (DF) (N = 88)**	**Over all p-value**	**R vs. D/C**	**R vs. DF**	**D/C vs. DF**
Age (mean, SD)	59.1 (12.3)	59.1 (9.9)	60.6 (14)	58.3 (12.9)	0.5944			
Male (%)	106 (54.9)	28 (47.5)	25 (54.4)	53 (60.2)	0.3112			
Distance from clinic (km)(median, IQR)	12.4 (7.4–20.5)	14.2 (7.9–20.7)	13.4 (7.2–17.8)	10.7 (7.5–23.9)	0.7194			
Urban (%)	174 (90.2)	51 (86.4)	45 (97.8)	78 (88.6)	0.1227	0.0741	0.6906	0.0967
Number of re-referrals (%)	36 (18.8)	14 (24.1%)	3 (6.5%)	19 (21.6%)	**0.0477**	**0.0158**	0.7188	0.0254
**Medical history**								
Duration DM2 (years) – (median, IQR)	6 (1–12)	8 (2–15)	2.5 (1–8.5)	7 (2.5–12)	**0.0187**	**0.0146**	0.7622	**0.0114**
Hypertension (%)	142 (74)	44 (74.6)	34 (75.6)	64 (72.7)	0.9321			
Dyslipidemia (%)	154 (83.7)	49 (86.0)	36 (83.7)	69 (82.1)	0.8338			
Psychiatric disease (%)	41 (21.9)	16 (27.6)	7 (15.6)	18 (21.4)	0.3388			
**Macrovascular complications**								
CAD (%)	41 (22.5)	16 (28.6)	8 (18.2)	17 (20.7)	0.4066			
PVD (%)	11 (6.0)	5 (8.5)	1 (2.3)	5 (6.1)	0.5275			
CVA/TIA (%)	10 (5.3)	6 (10.2)	0	4 (4.7)	0.0719	0.0380	0.3170	0.3005
Any macrovascular complications (%)	51 (28.3)	21 (36.8)	8 (18.6)	22 (27.5)	0.1311	0.0466	0.2455	0.2733
**Microvascular complications**								
Nephropathy (%)	49 (27.1)	18 (31.6)	9 (20.9)	22 (27.2)	0.4945			
Retinopathy (%)	26 (17.6)	12 (23.5)	2 (5.9)	12 (19.1)	0.1026	0.0316	0.5595	0.1281
Neuropathy (%)	49 (26.9)	13 (22.4)	7 (16.3)	29 (35.8)	**0.0424**	0.4443	0.0901	0.0226
Any microvascular complications (%)	79 (40.9)	24 (47.1)	13 (33.3)	42 (59.2)	**0.0328**	0.1897	0.1860	**0.0096**
**Disease status**								
HbA1c (%)(mean, SD)	8.5 (1.9)	8.6 (1.8)	7.9 (1.9)	8.7 (1.9)	0.0609	0.0669	0.6694	0.0243
HbA1c (IFCC units mmol/mol)	69	70	63	72				
HbA1c ≤7.0% or 53 mmol/mol (%)	47 (25.5)	16 (27.6)	17 (39.5)	14 (16.9)	**0.0199**	0.2055	0.1259	**0.0051**
Weight (kg) (mean, SD)	94.1 (23.2)	93.0 (22.3)	88.9 (18.9)	97.6 (25.3)	0.1122	0.3252	0.2608	0.0448
BMI (mean, SD)	33.8 (7.7)	33.8 (7.8)	32.5(6.3)	34.5 (8.2)	0.3738			
SBP (mean, SD)	140.9 (20.0)	141.2 (23.2)	138.7 (19.3)	141.8 (18.0)	0.6879			
DBP (mean, SD)	75.1 (10.0)	75.5 (11.1)	75.4 (9.4)	74.6 (9.5)	0.8267			
Total Cholesterol (mean, SD)	4.99 (1.41)	4.96 (1.44)	4.98 (1.1)	5.02 (1.55)	0.9694			
HDL-C (mean, SD)	1.19 (0.33)	1.22 (0.32)	1.21 (0.33)	1.15 (0.33)	0.5015			
LDL-C (mean, SD)	2.65 (1.01)	2.64 (1.04)	2.85 (1.03)	2.53 (0.97)	0.3139			
Triglycerides (median, IQR)	2.06 (1.5–2.92)	1.92 (1.39–2.99)	2.12 (1.58–2.59)	2.18 (1.5–2.99)	0.8033			
TC:HDL-C (mean, SD)	4.33 (1.41)	4.19 (1.27)	4.35 (1.27)	4.43 (1.59)	0.6311			
Smoker (%)	37 (20.1%)	8 (13.8)	7 (16.3)	22 (26.5)	0.1390	0.7283	0.0695	0.1960
**First visit medications**								
On Insulin (%)	47 (24.4%)	23 (39.0)	3 (6.5)	21 (23.9)	**0.0006**	**0.0001**	0.0498	**0.0129**
On ACE-I/ARB (%)	114 (59.7)	44 (74.6)	25 (54.4)	45 (52.3)	**0.0191**	0.0303	**0.0069**	0.8245
On ASA (%)	76 (39.8)	26 (44.1)	16 (35.6)	34 (39.1)	0.6684			
On Statin (%)	98 (50.8)	34 (57.6)	23 (50.0)	41 (46.6)	0.4198			

Note: p-values significant at 5%, with Bonferroni adjustment for pairwise comparisons, are printed in bold type. *R* Retained, *D/C* Discharged, *DF* Defaulted, *CAD* Coronary artery disease, *PVD* Peripheral vascular disease, *CVA* Cerebral vascular accident, *TIA* Transient ischemic attack, *HbA1c* Glycated hemoglobin, *kg* kilograms, *BMI* Body mass index, *SBP* Systolic blood pressure, *DBP* Diastolic blood pressure, *HDL-C* High-density lipoprotein-cholesterol, *TC:HDL-C* Total cholesterol to HDL-cholesterol ratio, *LDL-C* Low-density lipoprotein-cholesterol, *ACE-I* Angiotensin-converting enzyme inhibitor, *ARB* Angiotensin receptor blocker (antagonist), *ASA* Acetylsalicylic acid, *MD* Medical doctor, *RN* Registered nurse, *RD* Registered dietitian, *SD* Standard deviation, *IQR* Interquartile range.

Of the 193 patients, 29.6% (95% CI 24.1%-37.1%) were retained in clinic (after a median duration of follow-up of 33 months), 23.1% (95% CI 17.8%-29.9%) were discharged (after a median duration of follow-up of 10 months), and 44.2% (95% CI 38.6%-52.6%) defaulted (after a median duration of follow-up of 9 months).

When these 3 groups were examined retrospectively to their first visit at the FEDC (see Table 1), duration of diabetes, proportion of re-referrals, proportion of patients with any microvascular disease, neuropathy, insulin use, and HbA1c ≤7% (53 mmol/mol), were significantly different among the three groups based on the overall test of significance. Based on the pairwise comparisons, the retained group had longer duration of DM2, a higher proportion of re-referrals, and were more likely to be on insulin at baseline than the discharged group. Patients who had defaulted had longer duration of DM2, were more likely to have microvascular complications, were less likely to have HbA1c ≤7% and were more likely to be on insulin at baseline than those discharged. No difference was noted in mean distance travelled to the clinic or proportion living in an urban setting between groups.

A comparison of characteristics of the 3 groups at the last visit is presented in Table 2. Significant differences among the groups were found for the proportions of patients with any microvascular complications, retinopathy, and HbA1c ≤7%. The proportions of patients on insulin and acetylsalicylic acid (ASA) at last visit were also significantly different among the groups with the highest proportion on these medications in the retained group. Based on the pairwise comparisons, patients who defaulted were more likely to be on insulin and less likely to have HbA1c ≤7% at last visit than discharged patients. Compared with those retained in the clinic, defaulted patients were less likely to be on insulin or ASA at last visit. No significant differences among the groups were noted for macrovascular complications, blood pressure, smoking status, LDL-C, use of angiotensin-converting enzyme inhibitor (ACE-I), angiotensin receptor blocker antagonist (ARB) and use of statins.

**Table 2:** Comparison of last visit characteristics among patients retained, discharged, and defaulted

**Last visit characteristics**	**Overall (N = 193)**	**Retained (R) (N = 59)**	**Discharged (D/C) (N = 46)**	**Defaulted (DF) (N = 88)**	**Overall p-value**	**R vs. D/C**	**R vs. DF**	**D/C vs. DF**
**Macrovascular complications**								
CAD (%)	45 (23.8)	17 (29.3)	9 (20.0)	19 (22.1)	0.4802			
PVD (%)	14 (7.5)	6 (10.2)	1 (2.3)	7 (8.2)	0.3081			
CVA/TIA (%)	12 (6.3)	7 (12.1)	1 (2.2)	4 (4.6)	0.1305	0.1336	0.1164	0.4980
Any macrovascular complications (%)	57 (30.0)	23 (39.0)	10 (22.2)	24 (27.9)	0.1539			
**Microvascular complications**								
Nephropathy (%)	65 (35.7)	24 (40.7)	11 (25.6)	30 (37.5)	0.2635			
Retinopathy (%)	31 (18.8)	14 (25.6)	2 (5.1)	15 (21.7)	**0.0406**	**0.0121**	0.7080	0.0228
Neuropathy (%)	50 (27.3)	15 (25.9)	7 (15.9)	28 (34.6)	0.0785	0.2261	0.2735	0.0265
Any microvascular complications (%)	95 (53.7)	31 (53.5)	16 (37.2)	48 (63.2)	**0.0243**	0.1057	0.2576	**0.0064**
**Disease status (last visit):**								
HbA1c (%)(mean, SD)	7.6 (1.6)	7.8 (1.8)	6.6 (0.9)	7.9 (1.5)	**<0.0001**	**<0.0001**	0.7069	**<0.0001**
HbA1c (IFCC units mmol/mol)	60	62	49	63				
HbA1c ≤ 7.0% or 53 mmol/mol (%)	86 (46.2)	25 (42.4)	37 (84.1)	24 (28.9)	**<0.0001**	**<0.0001**	0.0964	**<0.0001**
Change in HbA1c (%) (mean, SD)	−0.90 (1.7)	−0.74 (1.75)	−1.28 (1.87)	−0.80 (1.69)	0.2480			
Weight (kg) (mean, SD)	94.5 (23.3)	94.3 (21.8)	88.8 (21.2)	97.6 (25.0)	0.1289	0.2074	0.4183	0.0495
Change in weight (mean, SD)	0.8 (5.1)	1.6 (5.6)	0.3 (5.3)	0.4 (4.7)	0.2978			
BMI	33.9 (7.7)	34.2 (7.4)	32.5 (7.2)	34.5 (8.2)	0.3726			
SBP (mean, SD)	139.2 (19.8)	141.7 (21.8)	133.4 (17.9)	140.5 (18.9)	0.0712	0.0390	0.7263	0.0376
DBP (mean, SD)	73.1 (9.6)	71.8 (9.6)	72.4 (10.4)	74.2 (9.1)	0.2828			
Total Chol (mean, SD)	4.5 (1.4)	4.4 (1.2)	4.4 (1.1)	4.7 (1.6)	0.2377			
HDL-C (mean, SD)	1.2 (0.3)	1.1 (0.3)	1.2 (0.3)	1.2 (0.3)	0.6053			
LDL-C (mean, SD)	2.3 (1.0)	2.4(1.0)	2.3 (1.0)	2.3 (1.0)	0.8984			
Triglycerides (median, QR)	1.8 (1.2–2.7)	1.7 (1.3–2.5)	1.8 (1.2–2.5)	1.9 (1.3–3.2)	0.3467			
TC:HDL-C (mean, SD)	4.1 (1.4)	4.03 (1.2)	3.82 (1.2)	4.31 (1.6)	0.1874			
Smoker (%)	33 (18.8)	6 (10.9)	7 (16.3)	20 (25.6)	0.0897	0.4367	0.0349	0.2365
**Last visit medications:**								
On insulin (%)	75 (38.9)	37 (62.7)	7 (15.2)	31 (35.2)	**<0.0001**	**<0.0001**	**0.0011**	**0.0147**
On ACE-I/ARB (%)	144 (74.6)	48 (81.4)	33 (71.7)	63 (71.6)	0.3604			
On ASA (%)	94 (48.7)	37 (62.7)	20 (43.5)	37 (42.1)	**0.0351**	0.0497	**0.0140**	0.8734
On Statin (%)	146 (75.7)	49 (83.1)	37 (80.4)	60 (68.2)	0.0825	0.7297	0.0436	0.1320
**Clinic activity**								
Median duration of follow-up (months)	16 (6 to 31)	33(31 to 35)	10 (4 to 18)	9 (5 to 17)	**<0.0001**	**<0.0001**	**<0.0001**	0.8827
% of MD visits where meds changed (Mean, SD)	48.7 (30.0)	55.2 (23.1)	34.4 (25.7)	51.8 (33.9)	**0.0007**	**<0.0001**	0.5037	**0.0027**
% of patient encounters that were no-shows (Median, IQR)	14.3 (0 to 33.3)	8.3 (0 to 16.7)	0 (0 to 14.3)	30 (13.8 to 50.0)	**<0.0001**	0.1112	**<0.0001**	**<0.0001**
Number of visits with MD (mean, SD)	4.9 (3.1)	8.1 (2.8)	3.3 (1.9)	3.7 (2.0)	**<0.0001**	**<0.0001**	**<0.0001**	0.2907
Number of visits with RN (mean, SD)	2.5 (2.1)	3.7 (2.6)	2.1 (1.7)	2.0 (1.7)	**<0.0001**	**0.0006**	**<0.0001**	0.6919
Number of visits with RD (mean, SD)	2.4 (2.0)	3.5 (2.5)	2.2 (1.9)	1.7 (1.3)	**<0.0001**	**0.0036**	**<0.0001**	0.1053

Note: p-values significant at 5%, with Bonferroni adjustment for pairwise comparisons, are printed in bold type, *R* Retained, *D/C* Discharged, *DF* Defaulted, *CAD* Coronary artery disease, *PVD* Peripheral vascular disease, *CVA* Cerebral vascular accident, *TIA* Transient ischemic attack, *HbA1c* Glycated hemoglobin, *kg* kilograms, *BMI* Body mass index, *SBP* Systolic blood pressure, *DBP* Diastolic blood pressure, *HDL-C* High-density lipoprotein-cholesterol, *TC:HDL-C* Total cholesterol to HDL-cholesterol ratio, *LDL-C* Low-density lipoprotein-cholesterol, *ACE-I* Angiotensin-converting enzyme inhibitor, *ARB* Angiotensin receptor blocker (antagonist), *ASA* Acetylsalicylic acid, *MD* Medical doctor, *RN* Registered nurse, *RD* Registered dietitian, *SD* Standard deviation, *IQR* Interquartile range.

Significant differences were noted for indicators of clinic activity (Table 2). The mean percentage of visits where medications were changed by the physician was lowest in the discharged group (34.4% versus 55.2% in the retained group and 51.8% in the defaulted group, p = 0.0007). The median percentage of missed appointments that were no-shows was highest in the defaulted group (30% versus 8.3% in the retained group and 0% in the discharged group, p < 0.0001).

**Figure 1:**
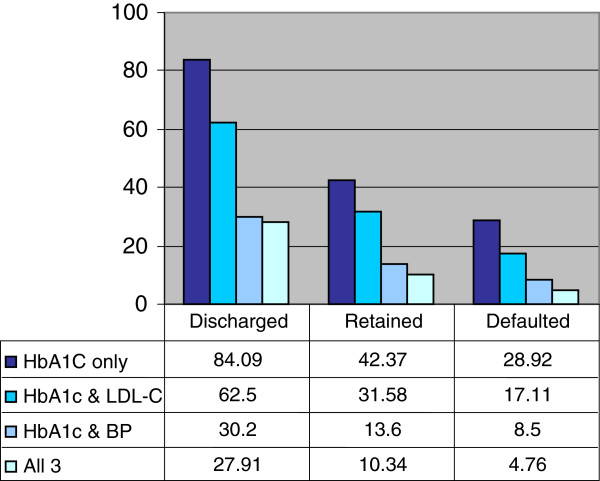
Percentage of patients in each group achieving 2003 Canadian Diabetes Association recommended targets for HbA1c, LDL-C and BP.

The percentages of patients meeting 2003 CDA Guideline recommended targets for HbA1c, LDL-C and BP at their last clinic visit are presented in Figure 1. The defaulted group had the lowest proportion of patients meeting all three targets at 4.8%. Of the patients who attained treatment targets for LDL-C and HbA1c, retained patients were more likely to be on insulin at their last clinic appointment (44.4%) than those discharged (12.0%) or defaulted (7.7%) but these differences were not statistically significant at the Bonferroni-corrected levels. There were no statistically significant differences in microvascular or macrovascular complications among groups in patients who had reached 2 or 3 targets at their last clinic visit.

Long-term outcomes of our cohort were available for 179 patients (Table 3). Patients without Ontario Health Card Numbers (e.g., residents of the neighbouring province of Quebec) (N = 14) were excluded from this analysis. A significantly lower proportion of patients who defaulted from tertiary care received recommended monitoring for their diabetes (HbA1c measurements, lipid measurements, and periodic eye examinations), despite no difference in mean number of visits to a primary care provider (PCP). The proportion of patients with emergency room visits was numerically higher in the defaulters group.

**Table 3:** Comparison of process-of-care indicators and outcomes 1 year after study end

**Variable**	**Retained N = 51**	**Discharged N = 45**	**Defaulted N = 83**	**P-value**
Mean A1c measurements received (sd)	1.92 (1.31)	1.58 (1.32)	1.37 (1.15)	0.055
Received A1C (%)	46 (90.2)	33 (73.3)	56 (67.5)	0.011
Received lipid profile (%)	46 (90.2)	35 (77.8)	54 (65.1)	0.004
Received eye exam (%)	41 (80.4)	27 (60.0)	44 (53.0)	0.006
Received Creatinine (%)	45 (88.2)	37 (82.2)	62 (74.7)	0.15
Mean visits to primary care (sd)	8 (7)	6.5 (5)	6.4 (6)	0.319
Received annual physical exam (%)	9 (17.6)	7 (15.6)	10 (12)	0.654
Proportion with an emergency room visit (%)	13 (25.5)	10 (22.2)	33 (39.8)	0.071
Proportion admitted to hospital (%)	9 (17.6)	6 (13.3)	18 (21.7)	0.501

## Discussion

We found almost half of the patients referred to the clinic (44.2%) defaulted from care, while 23.1% were discharged and 29.6% of patients were retained in clinic. Patients in the defaulted group had the lowest proportion reaching CDA treatment targets for HbA1c, LDL-C, and BP while attending diabetes clinic. One year after the last documented clinic appointment, the defaulted patient group also had the lowest proportion receiving A1C measurements, lipid profile measurements and eye examinations despite having a similar mean number of visits to primary care physicians as the discharged and retained patients groups. A possible explanation is primary care physicians may have been unaware that the patient had stopped attending diabetes clinic visits and were therefore not ordering diabetes monitoring investigations. Patients may also have perceived their diabetes as “less important” relative to their other medical problems and therefore did not follow through with recommended monitoring or the follow up with the diabetes clinic.

The long-term consequences of defaulting from a diabetes clinic such as worsened glucose control, increased BP, increased prevalence of complications, and increased all cause mortality have been reported [13]. Currie et al. have demonstrated a “dose –response” relationship with those having >2 missed appointments being at significantly higher risk of all cause mortality. Decreased monitoring may increase the need for emergency treatment of acute decompensations. The proportion of patients with emergency room visits was numerically higher in our cohort. Access to specialty care may be negatively impacted if these patients require re-referral to already overstretched diabetes resources. Identification of patients at risk of defaulting is necessary to both prevent negative impact on the health care system and possibly prevent adverse outcomes for patients. One finding that may signal a patient at risk of defaulting is failure to keep appointments [12,15]. Consistent with other groups, we also observed that the no-show rate was highest in the defaulted population.

The high proportion of patients defaulting from care was initially surprising to us. This rate is, however, similar to other reports [16]. The prevalence of defaulting from diabetes clinics ranges from 4-19% in Britain [10,17-19], 12-50% in the United States [11,20], and 35-57% in Japan [4,21]. In Canada, there are little data on defaulting rates. Shah et al. [22] reported that among patients referred to endocrinologists at 4 Canadian teaching hospitals, 23.5% did not return for a follow-up visit after an initial visit with the endocrinologist.

Our review was not designed to identify reasons for defaulting; however, we have identified some trends. Defaulters had a numerically higher mean weight, and the highest proportion of smokers. These findings were similar to those of Graber et al. [11] and Currie et al. [13], who found that the proportion of smokers was significantly higher in patients who missed clinic appointments. This finding may be due in part to the challenges patients who default from specialty care face in the management of their disease. These patients are less likely to participate in self-care, are more likely to have denial concerning their disease and are likely less engaged and empowered in the management of their disease. It is therefore not surprising that we observed that the defaulters group had the lowest proportion of patients meeting targets for HbA1c, BP, or LDL-C during the time they were followed in the diabetes clinic and were more likely to be on insulin than discharged patients. They had a similar percentage of visits where medications were changed by the attending physician to that of patients in the retained group [52% vs. 55%] suggesting unstable disease. These findings suggest that defaulters from our clinic had the most poorly controlled disease and should have had continued follow-up.

In contrast to our findings, Masuda et al. found defaulters were younger, less likely to be on medications, had shorter duration of diabetes, and lower HbA1c levels [23]. The authors concluded that these patients with “milder” disease may have been discharging themselves from clinic due to the belief that their disease was not severe enough to warrant intensive therapy. Simmons et al. and Currie et al. had similar results [13,24]. The differences between our population of defaulters and defaulters described by others likely reflect the referral pattern of our region in which the more complex patients with advanced disease are referred to tertiary care while patients with milder disease are cared for in the community. This finding may be similar to other communities with a similar referral structure.

It is also interesting to note that despite poor glycemic control, the percentage of patients on insulin at last visit with the FEDC was significantly lower in the defaulted group compared to the retained group. Some patients possibly chose not to return to clinic when insulin therapy was proposed. It is likely that patients who fail to achieve control because of inadequate adherence to behavioural change or medications are also likely to be inadherent with attending clinic [25].

The primary limitation of this study is the inherent nature of a chart audit. Patient records may be incomplete reflections of the care encounter. Lack of standardization in chart formats, variations in professional recording practices and legibility are also concerns [26]. Although we did not have the resources to conduct double extraction of the data, measures were taken to ensure inter-rater reliability. A data extraction guide was created and frequent meetings between auditors were held to minimize problems in interpretation of data recorded. Our analyses excluded patients with missing values, and to the extent that patients with missing values are significantly different from those included in the analysis, our comparisons may be biased.

## Conclusions

In our review of patients referred to an interdisciplinary clinic with DM2, we found a low discharge rate but a much higher than anticipated proportion of patients who defaulted from clinic. Patients who defaulted from clinic had the most uncontrolled disease and did not receive recommended monitoring of their diabetes after leaving the diabetes clinic despite continued visits to primary care. No show rates were highest in the defaulting population and could signal patients at risk of defaulting. Further study into the reasons, long-term consequences and strategies to prevent defaulting is required to ensure that this population receives the care they need.

## Competing interests

The authors declare they have no competing interests.

## Authors’ contributions

JCM conceived of the study, participated in study design, interpretation of data, and drafted the manuscript. JM conceived of the study, participated in study design, interpretation of data, data collection, and drafted the manuscript. BS participated in study design and performed statistical analysis. MT participated in study design and performed statistical analysis. EK participated in study design and interpretation of data. CT participated in study design, interpretation of data, data collection and helped to draft the manuscript. TO conceived of the study, participated in study design, interpretation of data and helped to draft the manuscript. CL participated in study design and was involved revising the manuscript. All authors read and approved the final manuscript.

## Pre-publication history

The pre-publication history for this paper can be accessed here:

http://www.biomedcentral.com/1472-6963/13/303/prepub
